# Baseline and Trend of Lymphocyte-to-Monocyte Ratio as Prognostic Factors in Epidermal Growth Factor Receptor Mutant Non-Small Cell Lung Cancer Patients Treated with First-Line Epidermal Growth Factor Receptor Tyrosine Kinase Inhibitors

**DOI:** 10.1371/journal.pone.0136252

**Published:** 2015-08-27

**Authors:** Yu-Mu Chen, Chien-Hao Lai, Huang-Chih Chang, Tung-Ying Chao, Chia-Cheng Tseng, Wen-Feng Fang, Chin-Chou Wang, Yu-Hsiu Chung, Yi-Hsi Wang, Mao-Chang Su, Kuo-Tung Huang, Hung-Chen Chen, Ya-Chun Chang, Meng-Chih Lin

**Affiliations:** 1 Division of Pulmonary and Critical Care Medicine, Department of Internal Medicine, Chang Gung Memorial Hospital-Kaohsiung Medical Center, Chang Gung University College of Medicine, Kaohsiung, Taiwan; 2 Department of Respiratory Care, Chang Gung Institute of Technology, Chiayi, Taiwan; Medical University of Graz, AUSTRIA

## Abstract

**Background:**

Patients with early-stage lung cancer who have a high baseline lymphocyte-to-monocyte ratio (LMR) have a favorable prognosis. However, the prognostic significance of LMR in patients with advanced-stage *EGFR*-mutant non-small cell lung cancer (NSCLC) receiving first-line epidermal growth factor receptor (EGFR)-tyrosine kinase inhibitors (TKIs) has not been established. We conducted a retrospective analysis to investigate the influence of LMR on clinical outcomes including progression-free survival (PFS) and overall survival (OS) in *EGFR*-mutant patients with NSCLC.

**Materials and Methods:**

Of 1310 lung cancer patients diagnosed between January 2011 and October 2013, 253 patients receiving first-line EGFR-TKIs for *EGFR*-mutant NSCLC were included. The cut-off values for baseline and the 1-month-to-baseline ratio of LMR (MBR), determined by using receiver operating characteristic curves, were 3.29 and 0.63, respectively. Patients were divided into 3 prognostic groups: high LMR and MBR, high LMR or MBR, and low LMR and MBR.

**Results:**

The mean patient age was 65.2 years, and 41% were men. The median PFS and OS were 10.3 and 22.0 months, respectively. The PFS in patients with high LMR and MBR, high LMR or MBR, and low LMR and MBR were 15.4, 7.1, and 2.0 months, respectively (p < 0.001), whereas the OS were 32.6, 13.7, and 5.1 months, respectively (p < 0.001).

**Conclusion:**

A combination of baseline and trend of LMR can be used to identify patients with a high mortality risk in *EGFR*-mutant NSCLC patients receiving first-line EGFR-TKIs.

## Introduction

Lung cancer is the leading cause of cancer-related death worldwide and in Taiwan, and the incidence of lung cancer in Taiwan is increasing.[1, 2] Epidermal growth factor receptor (EGFR) mutations are more common in Asian patients with non-small cell lung cancer (NSCLC) compared with non-Asians, in non-smokers compared with current or ex-smokers, and in adenocarcinoma compared with other cancer histologies.[3–5]

In *EGFR*-mutant NSCLC patients, EGFR-tyrosine kinase inhibitors (TKIs) can improve progression-free survival (PFS), overall survival (OS), and quality of life, and they are less toxic when compared with platinum-based doublet chemotherapy.[6–8]

Although presence of *EGFR* mutation is a robust predictor of EGFR-TKIs responsiveness, 17–29% of TKI-naïve patients do not respond to first-line TKIs.[9, 10] EGFR-TKIs response could be influenced by clinical characteristics; it is therefore reasonable to determine the significance of these characteristics, which might also affect patient survival.

Because lymphocytes play an important role in tumor eradication[11] and macrophages are associated with tumor progression[12, 13], we presumed that patients with higher lymphocyte-to-monocyte ratio (LMR) might have better prognosis in *EGFR*-mutant NSCLC patients receiving first-line EGFR-TKIs. The LMR was found to be a prognostic factor in hematological cancer [14, 15] and in several types of solid tumors. [16–18] In addition, elevated LMR was found to be an independent prognostic factor in patents with early-stage lung cancer after complete resection[19] and in patients with advanced-stage lung cancer who were undergoing platinum-based chemotherapies.[20] However, to the best of our knowledge, the prognostic significance of baseline and trend of LMR in *EGFR*-mutant NSCLC patients receiving first-line EGFR-TKIs has not been established. We conducted a retrospective analysis to investigate the influence of baseline and trend of LMR on PFS and OS.

## Material and Methods

### Patient and clinical characteristics

We conducted a retrospective study between January 2011 and October 2013 at Kaohsiung Chang Gung Memorial Hospital in Taiwan. Patients were followed-up until March 2015. Adult patients aged ≥18 years with histologically or cytologically confirmed stage IIIB or IV NSCLC with *EGFR* mutations who were undergoing first-line cancer therapy with EGFR–TKIs were included. Patients were excluded if they had received other chemotherapies, targeted therapy, or immunological therapies.

Baseline assessments including clinical parameters, hematological variables, biochemistry, chest radiography, chest computed tomography, bone scintigraphy, and brain magnetic resonance imaging were performed within 4 weeks of treatment initiation.

Clinical parameters included age, sex, smoking status, Eastern Cooperative Oncology Group (ECOG) performance status (PS), and history of diabetes mellitus. Hematological parameters included neutrophil, lymphocyte, and monocyte counts at baseline and 1-month after treatment initiation. Baseline LMR was obtained by dividing baseline lymphocyte count by monocyte count. The 1-month to baseline LMR (MBR) was obtained by dividing the 1-month LMR by the baseline LMR. This study was approved by the Institutional Review Board of Kaohsiung Chang Gung Memorial Hospital. The need for informed consent was waived.

### *EGFR* mutation testing

Tumor specimens were obtained by bronchoscopy, CT-guided biopsy, pleural effusion cytology, or surgical procedures. The *EGFR* mutational analyses was performed using SCORPIONS and ARMS polymerase chain reaction from fragments amplified from genomic DNA extracted from paraffin-embedded tissues (QIAGEN EGFR RGQ PCR KIT). Exon 19 deletion and L858R mutations were defined as common mutations. Other mutations or compound mutations were defined as uncommon mutations.

### EGFR-TKI treatment response evaluation

Patients underwent routine chest radiography every 2–4 weeks, and chest computed tomography every 2–3 months to evaluate tumor response. Disease progression was determined by the clinician according to Response Evaluation Criteria In Solid Tumors criteria 1.1 [21]. The primary endpoint was PFS defined as the first day of EGFR-TKI administration until disease progression, death before documented progression, or the last visit during the follow-up period. The secondary endpoint of OS was defined as the first day of EGFR-TKIs administration until death, loss to follow-up, or last follow-up.

### Statistical analyses

Statistical analyses were performed using MedCalc (version 14.10.2). Receiver operating characteristic (ROC) curves, Youden's index were used to determine the best cut-off value for LMR as a prognostic factor. PFS and OS analyses were performed using the Kaplan-Meier method and the log-rank test. Cox proportional hazards regression test were used to evaluate independent factors. Cox regression proportional hazard test were also used to determine continuous variables including lymphocyte count, monocyte count, baseline LMR, one month LMR, MBR and their association with PFS and OS. Spearman’s-Rho analysis was used to determine associations between LMR, clinical factors, PFS, and OS. Kruskal-Wallis test was used for assessing the relationship between LMR and ECOG PS. P value < 0.05 was considered significant in statistical tests.

## Results

### Patient characteristics

**Fig 1:**
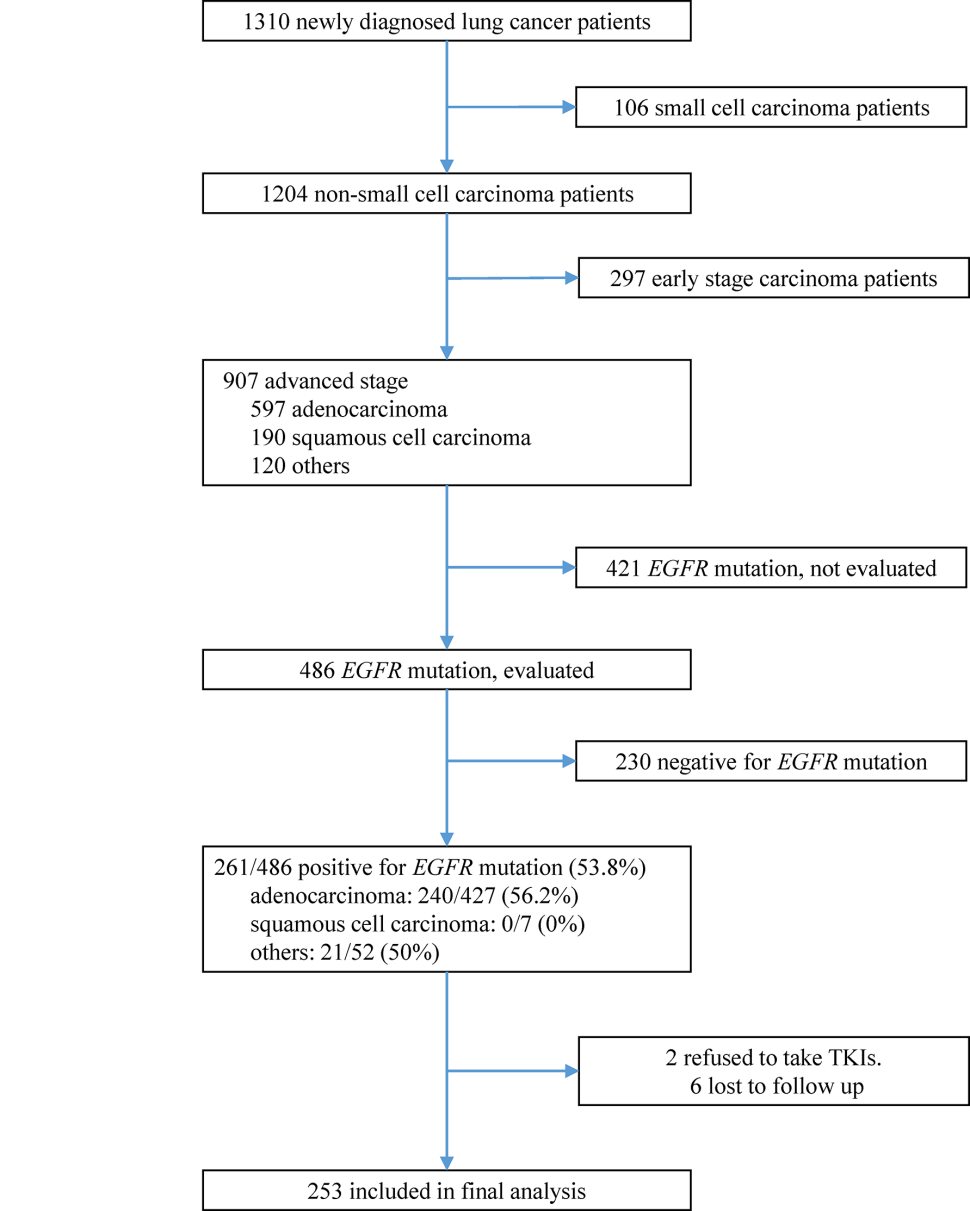
Inclusion, screening, and group assignment of patients. Among 1310 non-small-cell lung cancer patients diagnosed between January 2011 and October 2013, 253 patients were included into final analysis.

Among 1310 lung cancer patients diagnosed between January 2011 and October 2013, 486 patients with advanced NSCLC were screened for *EGFR* mutations (Fig 1). Of these, 261 (53.7%) patients had *EGFR*-mutant NSCLC. Two patients refused to undergo treatment with TKIs, and 6 patients were lost to follow-up. The final analysis data set consisted of 253 patients. All patients had baseline LMR data, and 1-month LMR data were available in 151 patients. The median follow-up time was 24.02 months, the longest follow-up time was 44.58 months. Clinical characteristics and therapy responses of all patients are shown in Table 1. At the last follow-up, 217 (85.8%) patients showed disease progression and 135 (53.4%) had died. The best cut-off point of LMR, MBR determined by ROC curve and Youden’s Index was 3.29, 0.63 respectively. Patients were divided into high or low LMR and MBR based on above cut-off value. There were 153 (60.5%) patients with high LMR, 100 patients (39.5%) with low LMR; 118 (78.1%) patients with high MBR and 33 (21.9%) patients with low MBR.

**Table 1:** Clinical characteristics and therapy responses of all 253 patients.

	Data (%)
Age (mean ± SD), years	65.2 ± 12.4
Sex	
Male	104 (41.1)
Female	149 (58.9)
DM	
Yes	48 (19.0)
No	205 (81.0)
Smoking history	
Non-smoker	172 (68.0)
Smoker	81 (32.0)
*EGFR* mutation ^a^	
Common	228 (90.1)
Uncommon	25 (9.9)
No of brain metastases	
0	196 (77.5)
1	14 (5.5)
2	8 (3.2)
>2	35 (13.8)
No of distant metastasis	
0–2	215 (85.0)
>2	38 (15.0)
Malignant effusion	
Yes	151 (60.1)
No	102 (39.9)
ECOG PS	
0–1	206 (81.4)
2–4	47 (18.6)
Lymphocyte (median ± IQR/mm^3^)	1599 ± 903e
Lymphocyte at 1 month (median ± IQR /mm^3^)	1397 ± 869
Monocyte (median ± IQR /mm^3^)	428 ± 279
Monocyte at 1 month (median ± IQR /mm^3^)	398 ± 278
LMR (median ± IQR)	3.6 ± 2.6
LMR at 1 month (median ± IQR)	3.2 ± 2.7
PFS (median), months	10.3
OS (median), months	22.0

^a^ Exon 19 deletion and L858R mutations were defined as common mutations. Other mutations or compound mutations were defined as uncommon mutations.

DM, diabetes mellitus; EGFR, epidermal growth factor receptor; ECOG, Eastern Cooperative Oncology Group; PS, performance status; LMR, lymphocyte-to-monocyte ratio; PFS, progression-free survival; OS, overall survival

### Survival analysis of clinical factors

**Fig 2:**
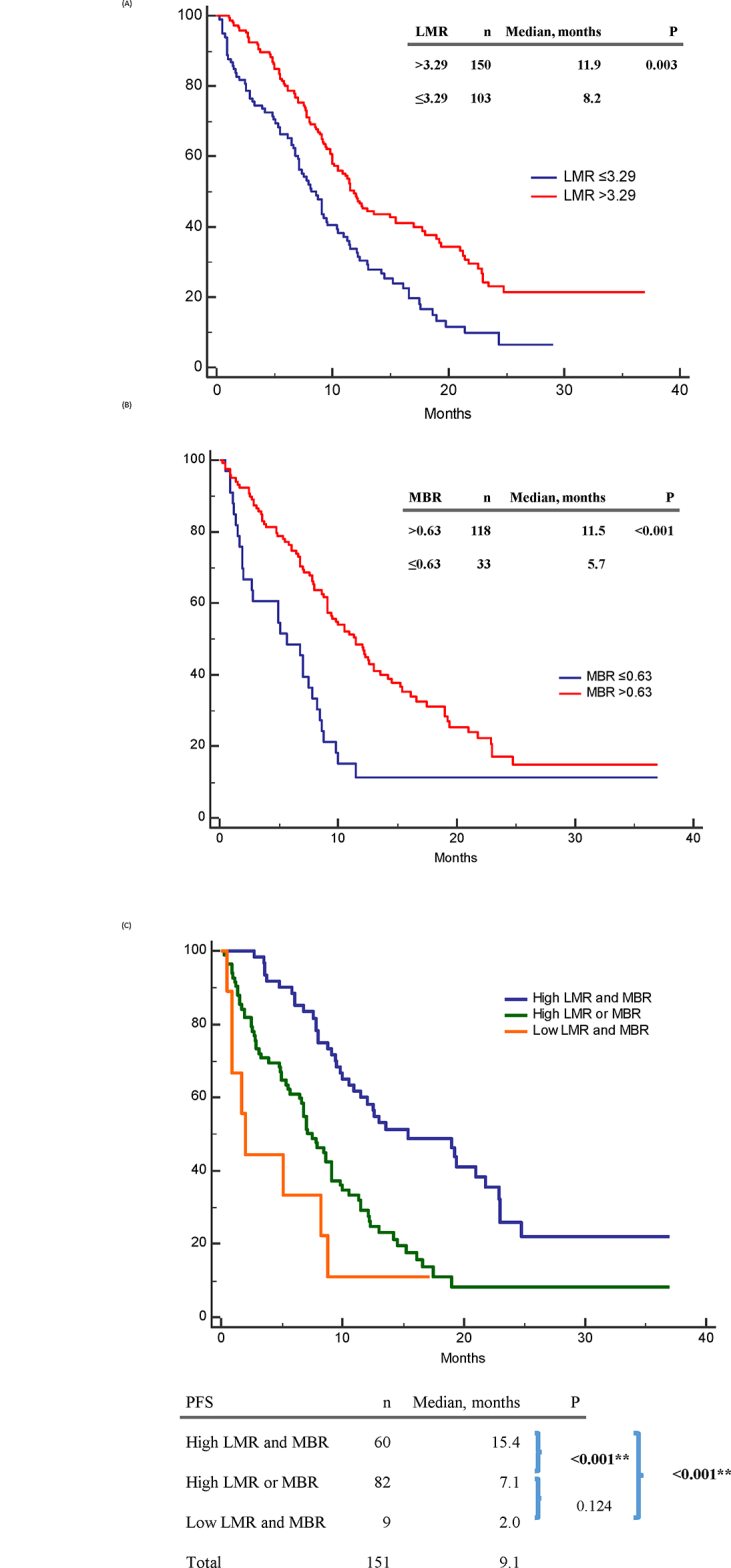
Progression-free survival (PFS) of epidermal growth factor receptor mutant non-small-cell lung cancer patients treated with first-line tyrosine kinase inhibitors therapy. (A) PFS between high and low baseline lymphocyte-to-monocyte ratio (LMR) patients; (B) PFS between high and low 1-month-to-baseline ratio of LMR (MBR) patients; (C) PFS between “high LMR and MBR”, “high LMR or MBR”, “low LMR and MBR” patients.

For PFS, clinical factors significant in univariable analysis included high LMR (p = 0.003) (Fig 2A), high MBR (p < 0.001) (Fig 2B), common *EGFR* mutations (p = 0.001), less distant organ metastases (p < 0.001), no malignant effusion (p = 0.007), and good ECOG PS (p < 0.001) (Table 2).

**Table 2:** Survival analysis of lymphocyte-to-monocyte ratio and clinical factors.

	PFS					OS				
	Univariable analysis		Multivariable analysis			Univariable analysis		Multivariable analysis		
	PFS	P	HR	95% CI	P	OS (m)	P	HR	95% CI	P
LMR		0.003		1.14–2.56	0.009		<0.001		1.66–3.35	<0.001
>3.29	11.9		1			33.8		1		
≤3.29	8.2		1.71			13.3		2.36		
MBR		<0.001		1.25–3.26	0.004		0.074			
>0.63	11.5		1			22.1				
≤0.63	5.7		2.01			12.9				
Age		0.124					0.461			
>65	11.5					17.8				
≤65	10.0					22.1				
Sex		0.631					0.254			
Male	10.4					18.4				
Female	11.1					23.0				
DM		0.061					0.925			
Yes	10.5					21.2				
No	10.5					23.0				
Smoking history		0.484					0.255			
Non-smoker	11.1					22.5				
Smoker	9.5					21.3				
EGFR Mutation^a^		0.001		1.06–3.45	0.032		0.286			
Common	11.3		1			21.4				
Uncommon	4.9		1.91			13.4				
Distant metastasis		<0.001		1.01–2.86	0.044		<0.001		1.41–3.27	<0.001
0–2	11.5		1			23.0		1		
>2	6.5		1.70			10.5		2.15		
Malignant effusion		0.007		0.75–1.63	0.599		0.010		0.98–1.97	0.065
Yes	9.2		1.11			17.5		1.39		
No	11.5		1			23.0		1		
PS		<0.001		0.97–2.37	0.071		<0.001		1.98–5.87	<0.001
ECOG 0–1	11.5		1			24.5		1		
ECOG 2–4	5.0		1.51			8.4		3.41		

^a^ Exon 19 deletion and L858R mutations were defined as common mutations. Other mutations or compound mutations were defined as uncommon mutations.

PFS, progression-free survival; OS, overall survival; LMR, lymphocyte-to-monocyte ratio; MBR, 1-month-to-baseline ratio of LMR; DM, diabetes mellitus; EGFR, epidermal growth factor receptor; PS, performance status; ECOG, Eastern Cooperative Oncology Group

Age, sex, DM history, smoking history, and tumor histology had no significant influence on PFS. In the multivariable analysis, independent predictive factors for a longer PFS were high LMR (p = 0.009), high MBR (p = 0.004), common *EGFR* mutations (p = 0.032), and having less distant organ metastases (p = 0.044) (Table 2.).

**Fig 3:**
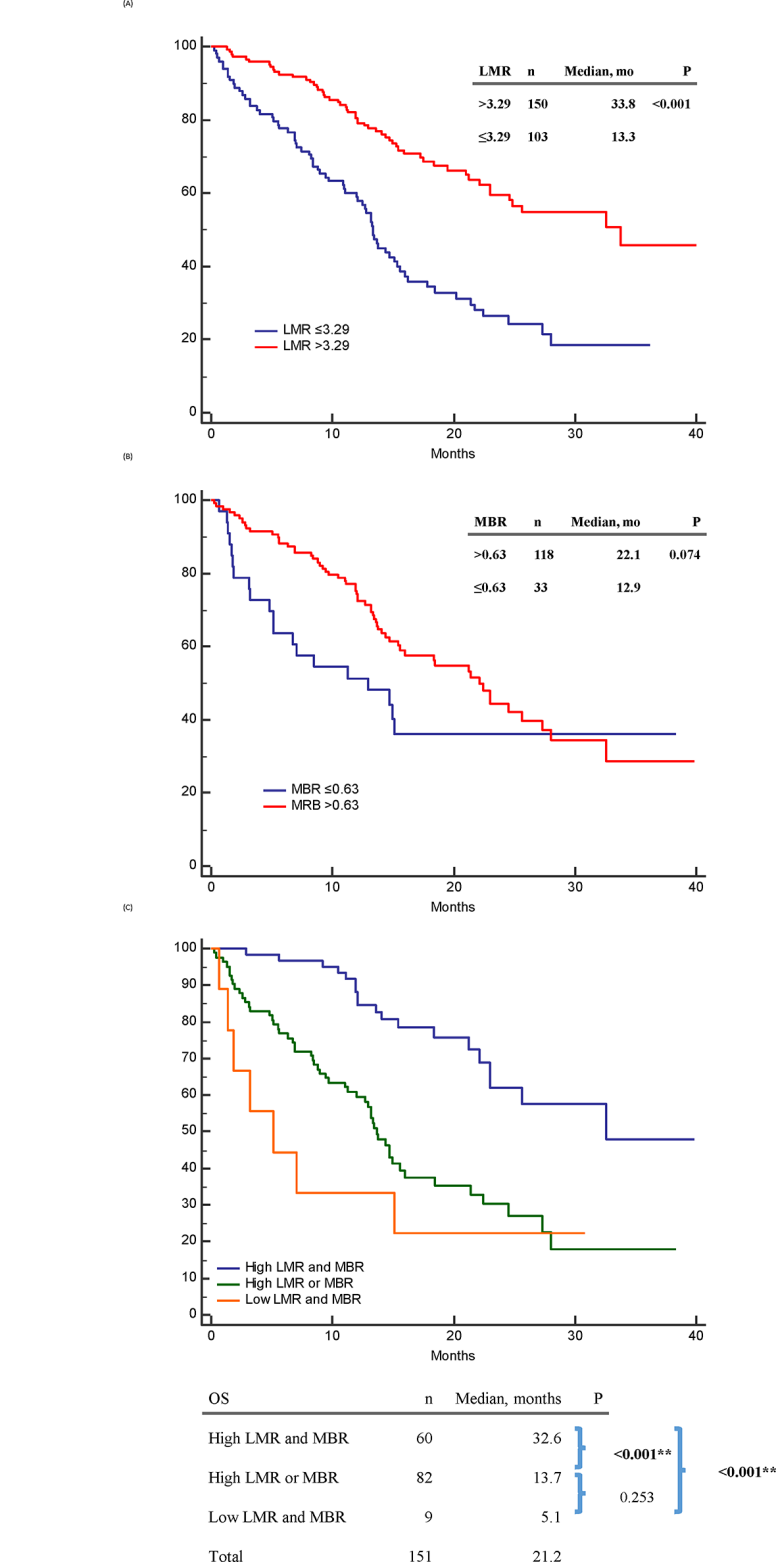
Overall survival (OS) of epidermal growth factor receptor mutant non-small-cell lung cancer patients treated with first-line tyrosine kinase inhibitors therapy. (A): OS between high and low baseline LMR patients; (B): OS between high and low 1-month-to-baseline ratio of LMR (MBR) patients; (C) OS between “high LMR and MBR”, “high LMR or MBR”, “low LMR and MBR” patients.

For OS, clinical factors significant in univariable analysis included high LMR (p < 0.001) (Fig 3A), less distant organ metastases (p < 0.001), no malignant effusion (p = 0.010), and good ECOG PS (p < 0.001). MBR (Fig 3B), age, sex, smoking history, and tumor histology had no significant influence on OS. In the multivariable analysis, high LMR (p < 0.001), less distant organ metastases (p < 0.001), and good ECOG PS (p < 0.001) were independent predictive factors for a longer OS.

### Combination of LMR and MBR for survival analysis

The PFS in patients with high LMR and MBR, high LMR or MBR, and low LMR and MBR were 15.4, 7.1, and 2.0 months, respectively (p < 0.001) (Fig 2C). The OS in the above three subgroups were 32.6, 13.7, and 5.1 months, respectively (p < 0.001) (Fig 3C).

### Correlations between LMR and clinical factors

**Fig 4:**
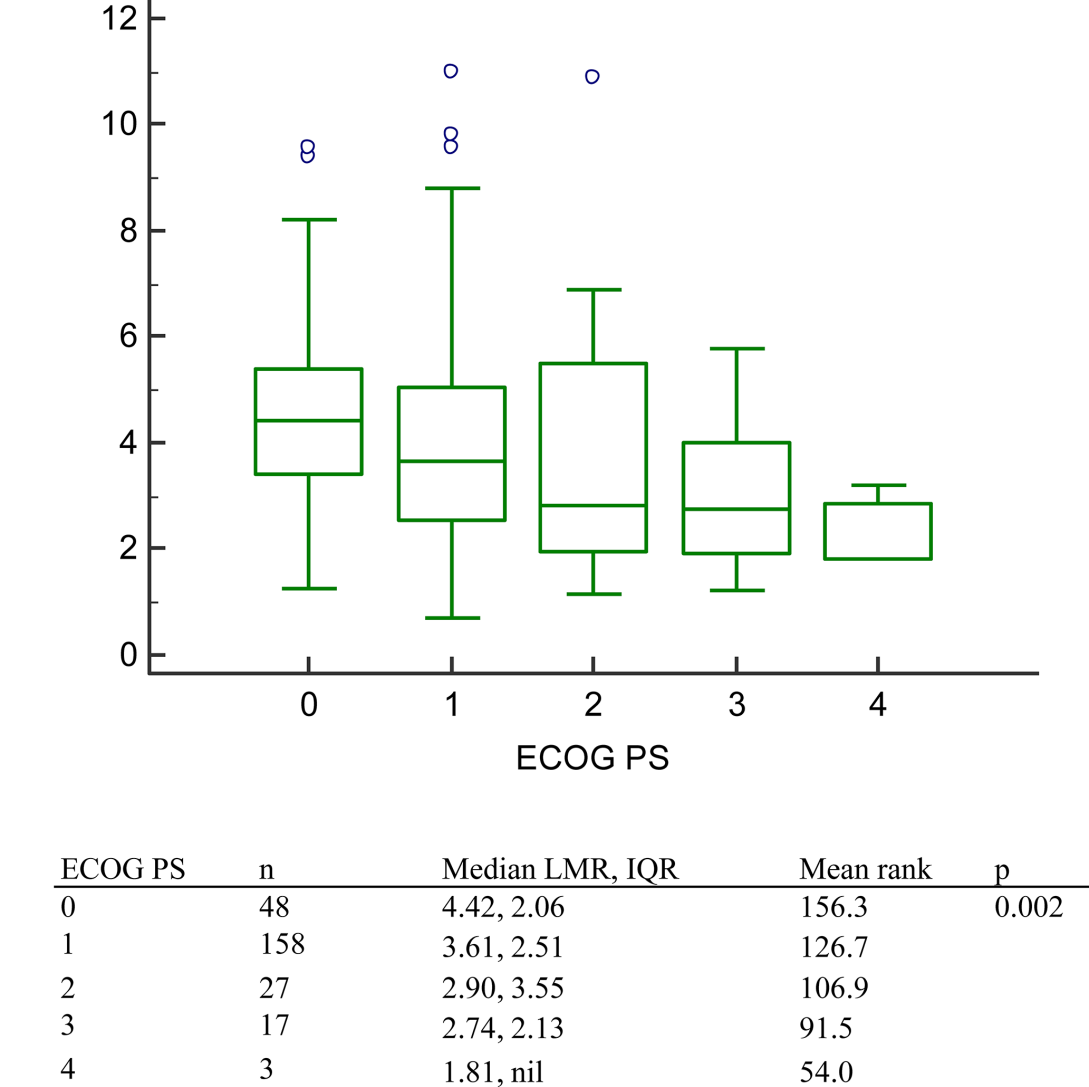
The lymphocyte-to-monocyte ratio in patients with different Eastern Cooperative Oncology Group performance status. Patients with better ECOG PS had higher Median LMR. (p = 0.002).

Significant correlations were noted between LMR and several clinical factors including the number of brain metastases, and the number of distant organ metastases. However, the correlation coefficient was low. The LMR correlation coefficients for the number of brain metastases, and the number of distant organ metastases were -0.147 (p = 0.020), and -0.209 (p = 0.001), respectively. (Table 3.) As to relationship between LMR and ECOG PS, significance was noted in Kruskal-Wallis test (p = 0.002). (Fig 4)

**Table 3:** Correlation between lymphocyte-to-monocyte ratio and clinical parameters.

	Correlation coefficient	P value
Age	-0.003	0.963
No of brain metastasis	-0.147	0.020
No. of distant metastasis organs	-0.209	0.001

## Discussion

Our study demonstrated that combination of LMR and MBR could predict prognosis in *EGFR*-mutant NSCLC patients receiving first-line EGFR-TKIs. Our study also revealed that a higher LMR correlated with a better ECOG PS, and a lower incidence of brain and distant metastases. Previous studies have demonstrated that Cytotoxic T lymphocytes play an important role in the anticancer response[11], and tumor-associated macrophages remodel the tumor extracellular matrix to promote proliferation, progression, and neovascularization[12, 13]. Based on the above pathophysiology, patients with a lower LMR most likely have a higher tumor burden and less cytotoxic T lymphocytes, which may, in part, explain why LMR acts as a prognostic factor in patients with advanced lung cancer who are receiving EGFR-TKIs. Besides being a prognostic factor, we hypothesize that peripheral blood LMR has the potential to act as a predictive factor for immunotherapy response because a low LMR would indicate less recruitable lymphocyte after the initiation of immunotherapy, although this concept requires further studies for confirmation.

A previous study indicated that LMR was a prognostic factor in hematological cancers such as diffuse large B-cell lymphoma, and postoperatively in several early-stage malignancies such as lung cancer[19], colorectal cancer[18], and non-metastatic nasopharyngeal carcinoma [16]. LMR has also been suggested as a prognostic factor in advanced-stage lung cancer patients receiving platinum-based chemotherapies[20], and in patients with breast cancer following neo-adjuvant chemotherapies.[17] To the best of our knowledge, this is the first study demonstrating that LMR is a prognostic factor in patients with advanced-stage, *EGFR*-mutant NSCLC receiving EGFR-TKIs.

Recently study revealed that CEA level change during EGFR-TKIs therapy can be used to a predictor of survival.[22] However, LMR change (MBR) during EGFR-TKIs was seldom mentioned before. Our study revealed patients with higher MBR had better PFS (11.5 vs 5.7 months, p < 0.001). Although patients with high MBR had almost twice longer OS than those with low MBR (22.1 vs. 12.9 months), no significant was noted in log-rank test. (p = 0.074) It needs more studies to prove if lack of significance in MBR on OS were due to small study population or a true negative result.

However, our study had several limitations. First, tumor programmed death-ligand 1 expression, which can provide direct information about the degree of immune paralysis in tumor microenvironment, was not available. Second, we do not have immune contexture, the amount of immune cell infiltration, in tumors, which can more precisely reflect the immune response in tumor microenvironment.[23] Further studies are required to determine whether add-on immunotherapy or anti-angiogenesis agents, in addition to first line EGFR-TKIs, could prolong survival in NSCLC patients, especially in those with low LMR. Finally, because our study was a retrospective study with a small patient population, a prospective trial is needed to validate these results.

## Conclusion

A combination of baseline and trend of LMR can be used to predict survival in *EGFR*-mutant NSCLC patients who treated with first-line EGFR-TKI therapy.

## Supporting Information

S1 Table Immune cell counts, ratios and their association with survival. As a continuous variable, monocyte count and lymphocyte-to-monocyte ratio had significant association with progression-free survival. Lymphocyte, monocyte count, and lymphocyte-to-monocyte ratio had significant association with overall survival.
(DOC)
