# Supplementary material for: Baseline and Trend of Lymphocyte-to-Monocyte Ratio as Prognostic Factors in Epidermal Growth Factor Receptor Mutant Non-Small Cell Lung Cancer Patients Treated with First-Line Epidermal Growth Factor Receptor Tyrosine Kinase Inhibitors
Source: PLoS One. 2015 Aug 27;10(8):e0136252. doi: 10.1371/journal.pone.0136252 (PMC4552380; doi:10.1371/journal.pone.0136252)
Supplement: S1 Table — As a continuous variable, monocyte count and lymphocyte-to-monocyte ratio had significant association with progression-free survival. Lymphocyte, monocyte count, and lymphocyte-to-monocyte ratio had significant association with overall survival. (DOC) [file pone.0136252.s001.doc]

|  | PFS | | OS | |
| --- | --- | --- | --- | --- |
|  | Hazard ratio | p | Hazard ratio | p |
| Lymphocyte count | 0.940 | 0.521 | 0.742 | 0.023 |
| Monocyte count | 2.309 | 0.001 | 3.436 | <0.001 |
| LMR | 0.895 | 0.005 | 0.761 | <0.001 |
| MBR | 0.999 | 0.986 | 1.077 | 0.190 |

**S1 Table.** **Immune cell counts, ratios and their association with survival.**

Abbreviations: PFS, progression-free survival; OS, overall survival; LMR, lymphocyte-to-monocyte; MBR, 1-month-to-baseline ratio of LMR.
